# Refugee healthcare needs and barriers to accessing healthcare services in New Zealand: a qualitative phenomenological approach

**DOI:** 10.1186/s12913-022-08560-8

**Published:** 2022-11-03

**Authors:** Bafreen Sherif, Ahmed Awaisu, Nadir Kheir

**Affiliations:** 1 Inpatient Pharmacy, Waitemata District Health Board, Auckland, New Zealand; 2 School of Pharmacy, Faculty of Medical and Health Sciences, Auckland University, Auckland, New Zealand; 3 College of Pharmacy, QU Health, Qatar University, Doha, Qatar; 4 College of Pharmacy, Ajman University, Al Jerf 2, P.O.Box 346, Ajman, United Arab Emirates

**Keywords:** Refugees and asylum seekers, New Zealand healthcare system, Healthcare access, Barriers to healthcare

## Abstract

**Background:**

Refuges and asylum seekers have specific healthcare needs; however there has been insufficient attention and effort to address these needs globally. Furthermore, effective communication between healthcare providers and refugees remains poor, further widening the imbalanced power dynamics. The aim of this research project was to examine refugee healthcare needs and current barriers to accessing healthcare services in New Zealand, and to propose solutions by exploring the perceptions, attitudes, beliefs, and opinions of key stakeholders regarding refugee healthcare needs within the scaffold of health and social care systems.

**Methods:**

We conducted semi-structured interviews between September and December 2018 with 18 purposively selected refugee service provider stakeholders in New Zealand using an interview guide that addressed healthcare needs, existing barriers to access healthcare services, and perceived future healthcare delivery solutions.

**Results:**

Thematic analysis of emergent themes of this study indicated the need for a national framework of inclusion, mandating cultural safety training of frontline personnel, improving access to interpreters and cultural mediators, and establishing the role of patient navigators. Barriers to accessing health services included entrenched social health determinants such as housing scarcity and disenfranchised community environments; refugee health-seeking behaviour and poor health literacy; along with existing social support networks. We propose that healthcare delivery should focus on capacity building of existing services, including co-design processes with refugees and asylum seekers and increasing funding for refugee-specific health service via the implementation of an overarching national strategy.

**Conclusion:**

Based on the findings of this study, refugee organisations and their frontline personnel should seek to address the deficiencies identified in order to provide equitable, timely and culturally-accessible healthcare services for refugees in New Zealand and in comparable countries.

## Background

There are an estimated 89.3 million forcibly displaced persons worldwide, the highest level documented, which includes 53.2 million internally displaced persons, 27.1 million refugees and 4.6 million asylum seekers owing to the fear of persecution or organised violence [1]. Refugees and asylum seekers are a heterogeneous cohort varying widely in education, health literacy, cultural beliefs, and behaviors [2, 3]. The dynamic process of the forced migration of refugee and asylum seeker populations contributes to added pressures on the health and social infrastructures of the receiving state, region or country and is ever evolving as a direct consequence of the ongoing global trends of persecution, conflict, violence or human rights violations. While forced migration itself is not a risk factor for poor health outcomes as migrants are often comparatively healthy, vulnerability to physical, mental and social health problems may arise from the process and specific circumstances of migration, giving rise to public health concerns [4–6]. The displacement of refugees and asylum seekers relates to public health issues including: exposure to hazards, propensity for communicable and non-communicable diseases (NCDs), re-emergence of neglected diseases, limited access to health services and intrinsic health-system barriers (cultural, social and linguistic), as well as health systems’ contingency planning [3].

The specific healthcare needs of refugees continues to be poorly understood globally and equally effective, tailored communication between healthcare providers and refugees remains poor. Additionally, there is often inadequate responsiveness of healthcare systems due to poor preparation, further amplified by medicolegal issues that refugees have to face with respect to access to health services. Approaches to managing refugee health problems or barriers to accessing health services have not sufficiently matched the pace of increasing challenges associated with the scale, diversity and disparity of current migration patterns [6–8].

Forced migration and the resultant creation of refugees is a top priority on the policy agendas of many of the world’s leading member states of the World Health Organisation (WHO). In the New Zealand setting, there is a trend for many services to evolve reactively to the arrival of migrants and refugees and are adapted to the perceived or expressed needs of the population [2, 3, 9]. The New Zealand Migrant Settlement and Integration Strategy was approved in 2014 on behalf of the Ministry of Business, Innovation and Employment (MBIE) and outlines the New Zealand government’s approach in response to the need for the integration of recently arrived migrants and refugees [10]. Refugees arriving in New Zealand may have long-term physical and psychological sequelae termed the ‘refugee experience’ encompassing the diverse physical and psychosocial experiences of refugees as they flee from conflict and persecution in search for safety.

### Hypothesis

This research stems from the realization that there is a paucity of data on the health status of refugees in New Zealand; and postulates that the current healthcare provision to refugees and their families is fragmented and may only be marginally addressing healthcare disparities and needs in this population [11].

### Aims

i) This research project examines a range of healthcare services that target refugees and the barriers to accessing these services as perceived by healthcare service providers in New Zealand to enable their elucidation.
ii) It also aims to propose refugee-centric solutions that enable increased accessibility of the refugee population to healthcare services by exploring the perceptions, attitudes, beliefs, and opinions in refugee healthcare needs of key stakeholders within health and social care systems.

## Theoretical/ conceptual framework

Qualitative research was utilised as it explores the stakeholders’ attitudes, values, beliefs, and opinions more fully [12]. An interview guide was developed by the primary author (BS) and revised by two research team members (NK, AA). The guide included open-ended, neutral questions covering three general domains comprising: current refugee healthcare needs, existing barriers to health access, and future healthcare delivery solutions.

Researcher reflexivity was attained by; (1) maintaining a research diary, (2) acknowledging the researcher’s existing feelings and experience with the phenomenon in question, and (3) explaining the influence of this experience on the data collection and analysis or findings and interpretation [6]. As migrants, the authors had first-hand knowledge of moving countries and cultures making reflexivity necessary. Lincoln and Guba’s evaluative criteria (1985) of credibility, transferability, dependability and confirmability were utilised to posit the trustworthiness research findings. As such, we implemented peer review processes allowing for a member checking step, described the research methodology in full, maintained a database of all research records and procedures and provided participants with a copy of the transcribed audio-recordings allowing an opportunity for edited transcripts thus achieving the credibility and dependability criterion [6].

Confirmability (i.e. that the study’s findings were based on the participants’ narratives rather than potential researcher biases) was attained through the maintenance of interview transcripts, describing the research procedures in full and utilising methods to enable future reproducibility and conducting a reflexivity analysis. Lastly, the extent to which the results attained can be applied in other contexts and studies (i.e. transferability) was ensured by recording and maintaining research-related outputs, and paying heed to the comprehensive presentation of data and discussion [6].

## Methods

### Eligibility criteria

To be included in the study, the potential participant had to be i) ≥ 18 years, ii) employed full-time for a minimum of one year with the respective refugee organisation and/or in the delivery of healthcare to refugees, iii) able to speak, read, and understand English, and iv) must have had direct contact with refugees. A total of 18 participants were recruited with experience in refugee health spanning from 2.1 to 8.3 years (Mean = 3.7 years). Participants were recruited from the public sector and encompassed healthcare providers, employees of NGOs and refugee health managers.

### Participants and sampling

Employees of purposefully selected organisations who met the eligibility criteria were invited to contact the primary researcher independently of their employers.

### Data collection

Semi-structured interviews were conducted via the modality preferable to participants with the same validated interview topic guide (Table 1) being utilized across the study participants allowing the elucidation of thoughts, opinions and perceptions of the stakeholders in keeping with an inductive thematic analysis methodology. Interview questions were focused around 3 main domains in keeping with the present study objectives of refugee healthcare needs, current barriers in accessing healthcare services and future healthcare direction. To this end, questions were asked about refugee choice of healthcare professional, how refugees come to understand healthcare services, the perceptions of healthcare workers towards traditional non-biomedical treatment modalities. Challenges faced in access to primary healthcare, access to pharmacist and medicines, cultural competence of existing services, quality of existing services accessed by refugees and the trajectory of refugee-centric healthcare delivery to name a few.

**Table 1:** Interview structure

Focus	Key Questions
Refugee healthcare needs	• Which healthcare professionals do refugees see most frequently? • How do refugees gain an understanding of health services? • What are some common health-related issues that refugees experience? • How supportive and responsive are health care delivery systems are towards refugee healthcare needs? • How accepting are we of refugee healthcare choices (e.g., the use of traditional healers/medicine)? • To what extent does the refugee experience in the country of origin affect the understanding of healthcare services on arrival?
Current barriers in accessing healthcare services	• Is there a reliance on accident and emergency services even where non-emergency treatment was appropriate? • What are some challenges faced by refugees in accessing primary healthcare in NZ? • How can we improve access to medicines and pharmacists? • To what extent do refugees under or over-report symptoms of ill health? • Do you think our health care system is adequately resourced to care for refugee patients and their families? • What in your opinion is a barrier to refugees attending a healthcare service? • Are there certain groups of refugees who are less likely to feel settled and/or have intrinsic barriers towards access to health? • To what extent do you believe our services are ‘culturally competent’? How does this translate across to refugee access to healthcare services? • Do you believe there is continuity of care across the different healthcare services?
Future healthcare direction	• Do you believe our healthcare services are responding sufficiently to perceived or expressed needs? • How do refugees feel about the quality of the services that they access? • How much influence do refugees believe they have in deciding the future direction of the services that they access? • What channels are there for refugees to provide feedback, and/or make suggestions about the direction of healthcare services? • Are healthcare services responding with enough momentum to refugee healthcare needs? • Do you foresee a role for pharmacists at the heart of health services that care for refugees? • How can pharmacists become involved in the refugee healthcare journey? • What do you believe are some barriers in refugees accessing pharmaceutical services? • How well do you believe refugees are integrating into New Zealand society? • What are your visions for the future direction of refugee healthcare in New Zealand?

Given the nature of phenomenological research, participants’ common and shared experiences were described through the use of semi-structured individual interviews which were conducted via the modality preferable to participants. All interviews were audio-recorded to enable edited transcribing and inductive analysis by the researchers with emphasis placed on understanding the content of the responses.

#### Procedure

Interviews were stopped when data saturation was deemed by the researchers to have been attained (i.e. similar views started to appear across interviews) at the conclusion of the eighteenth interview. As seen in Table 2, interviews were conducted with individuals across gender, age, occupations as well as several refugee organisations and resettlement centres in New Zealand, allowing for diversity and breadth of data.. All the interviews were conducted over a four-month period (Sept- Dec 2018) and each individual was interviewed once, with interviews averaging 30–40 minutes duration. To improve transparency and the quality of reporting in this qualitative research project, we used the Standards for Reporting Qualitative Research (SRQR) guidelines [12]. The SRQR provides succinct description of information required under six headings (Title and abstract, Introduction, Methods, Results/Findings, Discussion, Limitation, Others) with a total of 21 subheadings. All data was analysed using NviVO, version 12.0.

**Table 2:** Stakeholder’s characteristics (*n* = 18)

Sex	F: 15 (83%); M 3 (17%)
**Mean age in years**	45.3
**Occupation**	
**Nursing/Midwifery**	2
**Medical doctor**	6
**Staff in non-governmental organization**	3
**Translation services**	1
**Project Manager**	2
**Clinical Psychologist**	1
**Community Pharmacist**	2
**Research Fellow**	1
**Mean experience in refugee health in years**	3.7

### Analysis

Thematic content analysis was used to analyse the data generated from the interviews. This was operationalized through coding the edited transcripts using NVivo 12 for analysis, then generating categories from the coded terms or statements of interest in each transcript.

## Results

A total of 18 participants participated in the one-to-one interviews. Respondents came from diverse educational and vocational backgrounds (Table 2) ranging from those in clinical to non-clinical roles (nursing/midwifery:2, medical doctor:6, pharmacist: 2, clinical psychologist:1, NGO personnel: 3, translation services: 1, project management: 2, research fellow:1) and with a mean experience in refugee health of 3.7 years. Of the participants, 15 were female and 3 male. Only one participant identified as a previous refugee whereas 8 identified as immigrants to New Zealand.

### Thematic analysis results

**Fig. 1:**
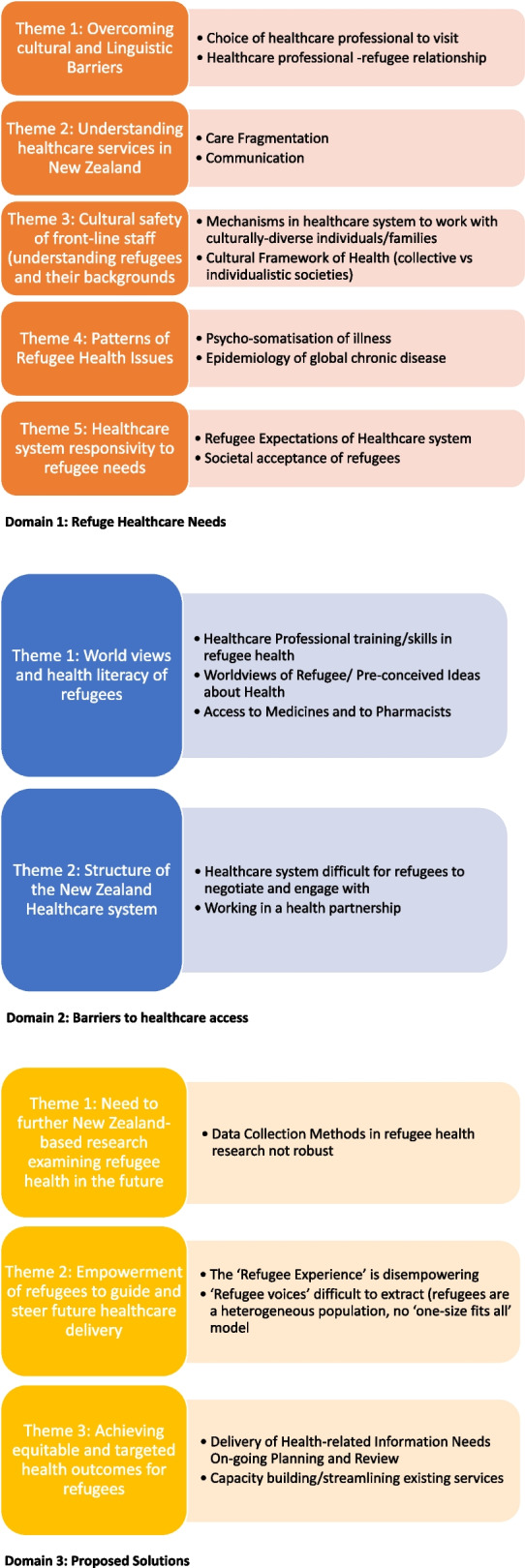
Summary of key themes emerging from the study findings

We have organised the findings by the 3 major predetermined domains and subsequent themes that emerged from the analysis of the interviews (Fig. 1). These are discussed in turn.

### Domain 1. Refugee healthcare needs

#### Overcoming cultural and linguistic barriers

i) *Choice of Health Professionals to Visit*

Gender and cultural background of healthcare professionals was perceived to be an important consideration when accessing healthcare. In general, refugees prefer to visit GPs from their own background as this ensures that both language and cultural barriers are overcome, even if this GP is not located in a geographically convenient location to the refugee(s). One of the participants commented that:

*“Some refugees prefer to go to a doctor from their background who speaks their language. Women, prefer women doctors.” P1.*

Gender preference seems to be much more complicated than a mere ‘preference’. Cultural paradigms and beliefs seemed often be much more important than personal safety. Women had particular needs around appropriate gender match for sensitive topics with this concerning both the interpreter and the medical professional involved. This was noted to be especially problematic in areas where female practitioners may not be readily available and requires pragmatic decision-making between the female refugee and the healthcare team.

ii) *Healthcare Professional-Refugee Relationship*

The dynamics of the healthcare professional-refugee relationship are such that there is a high risk of staff burnout. Refugees are perceived to be a high-needs population group requiring more intensive and frequent support from health professionals in the early stages of resettlement. Different health professionals may encounter refugees differently in the professional context as access of health professionals depends on the individual needs of the refugee. A strong element of trust is noted to favour the risk of staff burnout, as healthcare professionals are perceived to be more trustworthy and approachable. One of the participants explained:

*“Patients come to see a specific doctor not just for health needs but also for housing and employment - they trust the doctor more than just for health issues.” P2.*

An interviewee noted that it could be increasingly difficult to establish boundaries once the healthcare professional-refugee relationship has been well established. She goes on to say:

*“We have a translator onsite in the pharmacy and she is well-known and people will come see her. It is getting quite difficult for the interpreters because the community wants help and they can’t say no- they want to help but they have to have boundaries as well.” (P1).*

#### Understanding healthcare service delivery in New Zealand

i) *Care Fragmentation*

It was noted that the route of the refugee into the country plays an active role in their understanding of the New Zealand health service. There is the perception of a systematic delivery of healthcare and health services to quota refugees in New Zealand who undertake a structured resettlement program, whereas other categories of refugees are not granted the same privileges resulting in the fragmentation of healthcare delivery. One interviewee noted that for this latter group that it perhaps:

*“Depends on the primary healthcare service that they enrol with, and if they have lots of experience working with refugees.” (P12).*

Of note, the journey of a refugee is often long and protracted with elements of distrust of services being present. This factor is perceived by one GP as surmountable but time-dependent and requires persistence and effort on the part of the health service provider:

*“As they get to know you and trust you then they’ll tell you more things. Having on-going, continuity at one practice where they get to really know them is the best chance they have of disclosing things.”] (P6).*

This highlights the importance of building trust at the primary care level as a way of mitigating care fragmentation.

ii) *Communication*

A lack of understanding of health services and healthcare delivery systems may indirectly be demonstrated by potential or actual medication-related harm. Furthermore, the perceived increase range of medicines available in New Zealand highlights potential concerns where the safety and harm of medicines misuse is not communicated. This risk is of greater concern where language and health literacy barriers prevent the pharmacist from elucidating informed consent and understanding:

*“Being able to understand the dosages, why we are asking this and maximum doses in a day - they’ve never had the healthcare, they’ve never had the range of medicines so you have to be careful not to make assumptions.” P8.*

#### Cultural safety of frontline staff (understanding refugees and their background)

i) *Mechanisms in Healthcare System to Work with Culturally Diverse Individuals and Families*

A misunderstanding of refugees and their background on the part of service providers impedes healthcare access. This was perceived as a lack of adequate cultural safety. One participant commented:

*“We run cultural competency training for frontline staff annually, but because of staff turnover we’ve still got challenges- the terminology is becoming blurred and people are not understanding who is a refugee, who is a migrant, who is an asylum seeker.” P6*

ii) *Cultural Framework of Health (Collective* vs. *Individualistic Societies)*

Cultural paradigms were noted to influence acquisition of health-related knowledge with health professionals being encouraged to explore these views in detail.

*“There are different cultural ways in which people might see a particular health condition or the way that the community might deal with that. Health professionals need to understand that people come from different worldviews.” P12.*

#### Patterns of refugee health issues

i) *Psycho-somatization of Illnesses*

There was a perceived element of somatization of psychological distress which healthcare professionals should remain vigilant of.

*“A lot of people will have come from traumatic backgrounds and there may be a lot of psychological traumas that can manifest in a lot of pain in the body.” P8*

ii) *Epidemiology of Global Chronic Diseases*

A General Practitioner commented on the shift in the presentation of illnesses he has observed over the last decade which he attributes to a reduction of worldwide mortality and increased longevity making chronic disease more certain:

*“We are now dealing with chronic illness which we didn’t see before. People were coming mostly from refugee camps where there were limited medical facilities and people just died... We have gone from a population of infectious diseases and deficiencies to a population with chronic disease.” P5.*

#### Healthcare system responsiveness to refugee needs

i) *Refugee Expectations of Healthcare System*

Refugees may have unrealistic expectations and perceptions of the capability of the New Zealand’s health services particularly around the rules and regulations of medicines available over-the-counter without a prescription:

*“there is frustration at not being able to buy antibiotics and other medicines, the fact that GPs are gatekeepers and they’re very restricted about giving out antibiotics.” P6.*

Furthermore, it is thought that refugees may feel that health services are being unresponsive to their needs, but that this is often a case of high and/or unrealistic expectations. One GP noted:

*“Some people are expecting that they would see a specialist and have a CT or MRI scan- high intervention expectations.” (P9)*

ii) *Societal acceptance of refugees*

There appears to still be perceived difficulties with societal acceptance of refugees, but this not overshadowed by the fact that as a country New Zealand is thought to be faring comparatively well on the global stage. This was illustrated by a participant who suggested that.

*“most people feel welcomed and accepted but then people who are already disenfranchised and marginalized then go into communities that are in themselves disenfranchised and marginalized. There is that idea of a refugee coming in to take their houses, take their space- sadly they come and live in a world that propagates this idea of the working class or the poorest of the poor and ‘why don’t we look after our own first?” P3.*

Societal acceptance may relate to the social determinants of health with the host population feeling threatened by living and housing security, financial instability and the perceived neglect of one’s own vulnerable members of society at the expense of sheltering and caring for the vulnerable from overseas.

### Domain 2. Barriers to healthcare access

#### Worldviews and health literacy of refugees

i) *Healthcare Professional Training/Skills in Refugee Health*

Refugees experience barriers accessing healthcare services including perceptions of appropriate health-seeking behaviour with this being dependent on the variability of the individual health professionals’ training and expertise in refugee health. Refugee healthcare is perceived to be a niche area:

*“Within the refugee sector, the expertise and the experiences are quite good and getting better - outside of that, there is a lot of naivety within the society and community.” P13*

ii) *Worldviews of Refugees/Pre-conceived Ideas about Health*

A lack of engagement with GP services is attributed to the existing refugee worldviews and paradigms but may also be explained by New Zealand’s GP shortage that affects the entire population. An interviewee noted:

*“… for a lot of countries where these people come from; if they’re sick, they just go to the hospital.” P2*

iii) *Access to Medicines and to Pharmacists*

The diversity in the ethnic makeup of the pharmacist workforce is perceived as beneficial and other healthcare programs were advised to follow suit as a lack of ethnic and cultural diversity was perceived to be a barrier to healthcare access:

*“We have a much more diverse pharmacy workforce: it would be really good to get more students with former refugee backgrounds into health professional courses so that there is a range of different languages that’s able to be spoken.” P8.*

#### Structure of the New Zealand healthcare system

i) *Healthcare System Difficult for Refugees to Negotiate and Engage with*

Access to GP services for refugees is reasonable; however, primary care is perceived to be wider than the GP practice:

*“GP access is pretty good, but there is a whole range of other services and supports that mainstream people can access but unless somebody assists the refugee to that service, they’re probably not going to access it, and unless that service is equipped to meet their linguistic and cultural needs then they may not want to use it.” P1.*

Refugees require health navigation around these services while negotiating the maze-like primary care system. Refugees may perceive information sharing between services to be more streamlined than it actually is which may be related to a lack of understanding of the limitations of information sharing and of confidentiality rights. This in its basis may stem from differences in the refugee-preconceived model of health and that of our Western biomedical model.

ii) *Working in a health partnership*

Integrating refugee healthcare choices within New Zealand’s healthcare system was perceived to be dependent on the healthcare practitioner and their individual beliefs and understanding of the nuances in global healthcare practice. There was a perception that the successful integration and marriage of the at-times clashing ideologies was the basis of health partnership and informed decision-making. As noted by one participant:

*“It depends on how broad or wide your worldview is, how much you can understand the importance of other people’s worldviews. The biomedical view of health is arrogant in a sense because it’s based on the premise that there is a right and a wrong. If you’re working from that kind of theory then you’re not going to be very responsive to people’s choices. … It’s good to give them options, but you got to do that as a team.” P12.*

### Domain 3. Proposed solutions

#### Need for further New Zealand-based research examining refugee health in the future

i) *Data Collection Methods in Refugee Health Research Not Robust*

Services are engaged in consultations with refugee groups, but tangible changes are perceived as slow and this may relate to data collection methods for refugee health. There may be a necessity to change our data collection processes to enable the collection of tangible and refugee-centric data. The momentum of healthcare services’ response to refugee needs tends to be a slow process that is more localised to certain groups or community service providers, rather than on a regional/national level.

*“… there are endless consultations... it does really come down to how health stats are collected and reported … previously there was just one big Asian group, which statistically makes no sense. Now, many reports will have European, Maori, Pacific, and Asian and then other, but the MELAA (Middle eastern, Latin American and African) group, if you’re using that as a proxy for refugee, is often not reported.” P1.*

#### Empowerment of refugees to guide and steer future healthcare delivery

i) *The ‘Refugee Experience’ is disempowering*

People from refugee backgrounds working within health services tend to feel frustrated with the lack of opportunities for improvement of these services. A lack of improvement is often attributed to the difficulties in catering for the diverse and non- homogeneous refugee cohort within the financially under-resourced system:

*“The sorts of things that come back are ‘refugee groups are so small, we can’t do anything, there’s so many of them and they’re so diverse that we might as well not try to do anything to accommodate cultural needs.” P1.*

Furthermore, refugee clientele feel frustrated with on-going feedback and consultations processes, which are felt to be fruitless and increase the power distance between the refugee and the service. There was the perception that a strategic framework for inclusion and mandated cultural safety training for all health professionals should form our visions for the future. It is felt that refugee communities need to be involved first-hand in decision making, policy setting and in the provision of services directly to refugee communities.

ii) *‘Refugee Voices’ Difficult to Extract (Refugees are a Heterogeneous Population, no ‘One- size Fits All’ Model)*

Often, refugees not working immediately within the healthcare services are perceived to lack the realization that they have a ‘voice’ and can impact the future of services, which is an area that requires change:

*“I don’t think most refugees see themselves as being people that can influence the health system - they actually need to realise that their voices are important.” P10.*

There was a perception that the lack of volition of the refugee to provide feedback is related to the power-distance between the healthcare practitioner, and a lack of confidence in their rights to do so.

#### Achieving equitable targeted health outcomes for refugees

i) *Delivery of Health-related Information Requires On-going Planning and Review*

Establishment of a relationship and rapport between the pharmacist, the refugee client and the healthcare system is seen as pivotal to effective health education. There was a perceived need to improve the integration of community pharmacies within the primary healthcare model and to recognise pharmacists as valuable members of the multi-disciplinary health professional team in being able to strengthen communication ties.

*“Pharmacists can deliver training and education sessions for refugees - arrange workshops, meetings, and information sessions to explain the role of pharmacist and also how pharmacists can be helpful to them in the community.” P4.*

There was also the competing perception however, that a drive to increase community pharmacy services may be a detriment to refugee clientele as it poses the risk of care fragmentation.

ii) *Capacity Building/Streamlining Existing Services*

Healthcare services make efforts to respond to the needs of refugees but at times these needs fall outside the realm of expertise of staff and/or may occur too slowly for there to be quantifiable change:

*“..some of the health conditions are quite unique to certain groups. For example, female genital mutilation (FGM) - it’s practiced around the world in many countries and there are some nuances of these which we are not familiar with.” P2.*

## Discussion

This study draws on national and international literature as a comparison. It is recognised and acknowledge that much of the refugee health research conducted in New Zealand has been on a small scale and locally- based. Therefore, the findings have limited generalizability to all refugee/migrant populations and all health issues [13].

Clearly to address healthcare access barriers, there is a need for translation services in primary healthcare, information about the New Zealand health system and pertinent health information to be made available in ethnic minority languages [10, 13]. This aspect has been emphasized multiple times by several participants this study. Gil-Salmerón et al., (2021) noted that discrimination based on ethnicity and a lack of translation services in healthcare have been identified as main barriers to healthcare access. A total of 1407 migrants across 10 European Union countries were surveyed concerning healthcare discrimination, access to healthcare services, and need of translation services using an interviewed-administered questionnaire. The authors concluded that migrant and refugee patients reported unequal access to healthcare and perceived discrimination when they did access services; language communication support and cultural mediation in healthcare services were noted to facilitate healthcare access [5].

Cultural safety training for the healthcare workforce and capacity building in mainstream services are needed rather than establishing separate ethnic-specific services. Furthermore, there is a need to include refugee and migrant groups in both the national and regional health policy and strategy and to standardise ethnic data collection systems in a manner that allows the recognition of ethnic minority groups in New Zealand. There is also an on-going need to improve migrant and refugee research given the extensive gaps in research and information about refugee and migrant population health including a need for longitudinal data on the health of refugee/migrant populations in New Zealand.

Akin to the findings of Pavli et al. (2017), this research also noted that communication gaps between healthcare providers and refugees exist, creating difficulties in clearly elucidating the specific health needs of refugees [7, 8, 14]. Additionally, there is often an inadequate response of healthcare systems to these needs due to poor preparedness and/or fragmentation of care. Refugees are perceived to be a high-needs population group requiring more intensive and frequent support from healthcare professionals. Barriers to accessing healthcare services by refugees still exist, though efforts have been made in the larger centres such as Auckland to increase consultation times through refugee wrap-around services. Approaches to managing refugee health problems need to contend with the increasing challenges associated with healthcare delivery to a heterogeneous refugee population that is growing in size and displays a diversity and disparity of healthcare needs and unique barriers to access [6, 8, 10, 13, 15].

Effective healthcare provision may be impeded by the lack of healthcare professionals with the required experience of working with refugees. The lack of appreciation of the distinction between terminologies (migrant versus refugee) intersects with the perceived existence of discrimination; whether that be subconscious and internalized racism or conscious racism, highlighting the on-going need for cultural safety training of front-line staff in order to maintain practices and standards that culturally and linguistically diverse individuals such as refugees find acceptable. The western biomedical health model is perceived to be too rigid and healthcare professionals should be encouraged to adopt a more holistic and patient-centred approach when working with refugee clientele. Healthcare professionals would benefit from on-going education about refugees as part of cultural safety.

Refugees often have a collective attitude to healthcare and have a need for readily accessible interpreters, culturally competent and sensitive health practitioners and health information that is translated and made available in all the common refugee languages. Of note, collective attitudes may clash with the western individualistic system and can be ethically challenging for health practitioners requiring a collaborative and culturally sensitive approach.

Healthcare practitioners are perceived to have an important role in untangling and deconstructing the narrative motivating refugee healthcare choices in a non-judgmental and non-paternalistic manner to optimise care for the refugee client. Furthermore, there was an emphasis on the need for health professionals to resist dictating or guiding the refugee client and their families towards one preferred treatment modality as to do so would go against the spirit of partnership in healthcare.

Pharmacists are noted to be frontline healthcare professionals and have a role as facilitators in the refugee health journey. The on-going cultural safety requirement of pharmacists is viewed positively towards bettering access for minority groups. It was noted that cultural safety is best embedded within the framework of appropriate communication modalities available for use in pharmacies. The costs associated with accessing pharmaceutical services are perceived to be a deterrent for refugees although the advent of nil prescription co-payment pharmacies to mitigate this issue was recognized, but not ethically endorsed nor promoted. Refugees may have access to pharmacies and pharmacists but not necessarily access to the understanding of medicines-related knowledge and this is partly related to their health literacy. Nonetheless, pharmacists should be proactive in seeking access to phone translators where necessary and not making assumptions of a refugee client’s ability to comprehend health-related information:

The potential role of primary care in reducing inequity of access and of raising the quality of care should be fully explored, and the use of patient health navigation is recommended in advocating for refugees as is utilizing the role of community pharmacies as healthcare partners in assisting with individual refugee access, and in the delivery of community education about the New Zealand healthcare system.

Patterns of refugee health issues have evolved over the last decade with a paradigm shift away from acute illnesses towards that of chronic diseases. This phenomenon mirrors that of the host population and is not unique to the New Zealand refugee context but is a worldwide trend. Care and expertise to identify and refer symptoms that are psychosomatic in nature early in the treatment course to appropriate support services is crucial. The mental health of refugees is perceived to exist on a continuum and not all refugees will require referral to mental health services, but staff should be cognizant that underreporting of symptoms and or enveloping symptoms within the context of a physical ailment may be possible. Of note, refugee resilience factors and successful resettlement are viewed to be protective factors and it is important that healthcare professionals identify and differentiate between normal anxiety and clinically relevant anxiety and psychopathology.

Refugees may be under-reporting symptoms due to other competing interests taking precedence and/or fear of jeopardising their immigration status. Conversely, over-reporting of symptoms also increases in likelihood where there is the presence of trust and rapport between the healthcare provider and the refugee. While refugees are able to build this trust with healthcare providers, this often takes time and technique, which not all healthcare providers may be equipped with. Interestingly, a different viewpoint was offered in that refugees may simply be perceived as under-reporting or over-reporting by the healthcare provider whereas in actual fact there may be a misunderstanding of the different roles of healthcare providers which may be modifying reporting behaviour.

This research supports collateral, multi-organisational efforts that pool existing resources in the most culturally appropriate manner under the umbrella of an overarching government-led national refugee framework with a focus on health equity. Refugees often have a collective attitude to healthcare and have a need for readily accessible interpreters, culturally competent healthcare practitioners and health information that is translated and made available in all the common refugee languages. Refugees want to feel welcomed, listened to and respected at all points of healthcare access. Granting refugees access to culturally and linguistically appropriate health services and encouraging integration to the host society is fundamental to ensuring the collective health security of the country. There was also the perception of a need to develop an encapsulating refugee framework as part of service coordination. Improving service coordination on a regional level has long-term benefits in reducing the influx of refugees away from the smaller resettlement centres to the larger cities.

This study reports the experiences of refugees and asylum seekers between 2018 and 2019. The inequalities and vulnerabilities demonstrated in this study may have been further amplified by the COVID-19 pandemic which was not captured by our study. Whilst healthcare services face an escalating and unprecedented demand in services, epidemiological data collected in New Zealand has demonstrated that COVID-19 has disproportionately affected those with chronic conditions and underlying comorbidities as well as ethnic minorities and those living in areas of socioeconomic deprivation [16].

The findings of this study suggest a more proactive and refugee-responsive public health system as one where its national ethnicity system is able to epidemiologically categorise and identify refugees in a population. This research highlights the limitations around how New Zealand refugee health data is gathered and maintained, on a regional and national level, and recommends a review of refugee health information and ethnicity classification as part of the on-going scope for future refugee healthcare direction. Furthermore, there is a lack of a central data repository that compiles and maintains refugee health data obtained during the domestic health screenings which fragments care for refugees who may be enrolled with a GP but not necessarily heavily engaged with the GP services and favour attendance of emergency and Accident and Emergency services. Due to a lack of robust centralization of data, population-specific risk factor information is not easily recognised or retained. Therefore, policymakers need to strengthen the reporting process by creating a centralized system that recognises and retains refugee population-specific risk factor information relevant to the New Zealand and global context. We support the need for the creation of an overarching New Zealand-specific framework for addressing cultural diversity and for policy and funding strategies to recognise health needs in refugee groups.

## Conclusion

The present research examines refugee healthcare needs and current barriers to accessing healthcare services and proffers refugee-centric solutions to increase accessibility of the refugee population to healthcare services. Interviewees indicated the need for a national framework of inclusion, mandating cultural safety training of frontline personnel, improving access to interpreters and cultural mediators and establishing the role of patient navigators. Barriers to accessing health services includes existing social health determinants such as housing scarcity and disenfranchised community environments; refugee health-seeking behaviour and health literacy; along with existing social support networks. It is proposed that healthcare delivery should focus on capacity building of existing services, including co-design processes with refugees and asylum seekers and increasing funding for refugee-specific health service via the implementation of national approach.

## Data Availability

The datasets used and/or analysed during the current study are available from the corresponding author on reasonable request.

## Abbreviations

FGM: Female genital mutilation
SRQR: Standards for reporting qualitative research
UAHPEC: University of Auckland Human Participants Ethics Committee
